# Tramadol hydro­chloride–benzoic acid (1/1)

**DOI:** 10.1107/S1600536811032181

**Published:** 2011-08-17

**Authors:** B. P. Siddaraju, Jerry P. Jasinski, James A. Golen, H. S. Yathirajan, C. R. Raju

**Affiliations:** a Department of Studies in Chemistry, University of Mysore, Manasagangotri, Mysore 570 006, India; b Department of Chemistry, Keene State College, 229 Main Street, Keene, NH 03435-2001, USA; c Department of Chemistry, PES College of Science, Mandya, 571 401, India

## Abstract

In the cation of the title co-crystal salt {systematic name: [2-hydroxy-2-(3-meth­oxy­phen­yl)cyclo­hexyl­meth­yl]dimethyl­aza­nium chloride–benzoic acid (1/1)}, C_16_H_31_NO_2_^+^·Cl^−^·C_7_H_6_O_2_, the N atom is protonated and the six-membered cyclo­hexane ring adopts a slightly distorted chair conformation. The dihedral angle between the mean planes of the benzene rings in the cation and the benzoic acid mol­ecule is 75.5 (9)°. The crystal packing is stabilized by weak inter­molecular O—H⋯Cl, N—H⋯Cl and C—H⋯π inter­actions, forming a two-dimensional chain network along the *b* axis. The benzoic acid mol­ecule is not involved in the usual head-to-tail dimer bonding, but instead is linked to the ammonium cation through mutual hydrogen-bonding inter­actions with the chloride anion.

## Related literature

For the use of tramadol for perioperative pain relief, see: Scott & Perry (2000 ▶). For related structures, see: Arman *et al.* (2010 ▶); Hemamalini & Fun (2010 ▶); Tessler & Goldberg (2004 ▶). For standard bond lengths, see Allen *et al.* (1987 ▶).
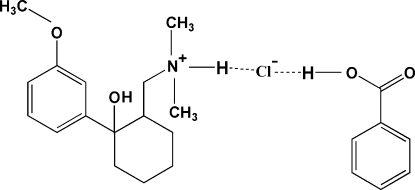


## Experimental

### 

#### Crystal data

C_16_H_26_NO_2_^+^·Cl^−^·C_7_H_6_O_2_
*M* *_r_* = 421.95
Monoclinic, 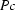

*a* = 8.9721 (2) Å
*b* = 10.4086 (2) Å
*c* = 12.5189 (3) Å
β = 101.646 (2)°
*V* = 1145.03 (4) Å^3^
*Z* = 2
Mo *K*α radiation
μ = 0.19 mm^−1^
*T* = 173 K
0.42 × 0.34 × 0.25 mm

#### Data collection

Oxford Diffraction Xcalibur Eos Gemini diffractometer
Absorption correction: multi-scan (*CrysAlis RED*; Oxford Diffraction, 2010 ▶) *T*_min_ = 0.923, *T*_max_ = 0.953
11101 measured reflections
5354 independent reflections
5067 reflections with *I* > 2σ(*I*)
*R*_int_ = 0.014

#### Refinement

*R*[*F* ^2^ > 2σ(*F* ^2^)] = 0.029
*wR*(*F* ^2^) = 0.077
*S* = 1.04
5354 reflections
267 parameters
2 restraints
H-atom parameters constrained
Δρ_max_ = 0.21 e Å^−3^
Δρ_min_ = −0.14 e Å^−3^
Absolute structure: Flack (1983 ▶), 2397 Friedel pairs
Flack parameter: −0.02 (4)

### 

Data collection: *CrysAlis PRO* (Oxford Diffraction, 2010 ▶); cell refinement: *CrysAlis PRO*; data reduction: *CrysAlis RED* (Oxford Diffraction, 2010 ▶); program(s) used to solve structure: *SHELXS97* (Sheldrick, 2008 ▶); program(s) used to refine structure: *SHELXL97* (Sheldrick, 2008 ▶); molecular graphics: *SHELXTL* (Sheldrick, 2008 ▶); software used to prepare material for publication: *SHELXTL*.

## Supplementary Material

Crystal structure: contains datablock(s) global, I. DOI: 10.1107/S1600536811032181/bv2189sup1.cif

Structure factors: contains datablock(s) I. DOI: 10.1107/S1600536811032181/bv2189Isup2.hkl

Supplementary material file. DOI: 10.1107/S1600536811032181/bv2189Isup3.cml

Additional supplementary materials:  crystallographic information; 3D view; checkCIF report

## Figures and Tables

**Table 1:** Hydrogen-bond geometry (Å, °) *Cg*3 is the centroid of the C18–C23 ring.

*D*—H⋯*A*	*D*—H	H⋯*A*	*D*⋯*A*	*D*—H⋯*A*
O1—H1*O*⋯Cl1^i^	0.84	2.36	3.1898 (9)	171
O3—H3*O*⋯Cl1	0.84	2.24	3.0620 (14)	167
N1—H1*N*⋯Cl1	0.93	2.21	3.0750 (12)	155
C1—H1*B*⋯*Cg*3^ii^	0.98	2.90	3.662 (5)	135

Symmetry codes: (i) 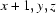; (ii) 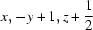.

**Fig. 1.**
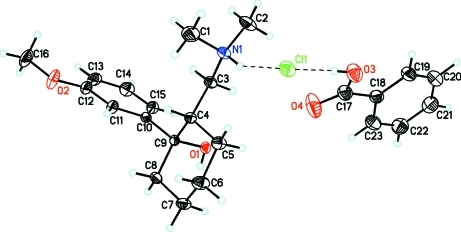
Molecular structure of the title co-crystal salt showing the atom labeling scheme and 30% probability displacement ellipsoids. The dashed lines represent weak O—H···Cl and N—H···Cl intermolecular interactions.

**Fig. 2.**
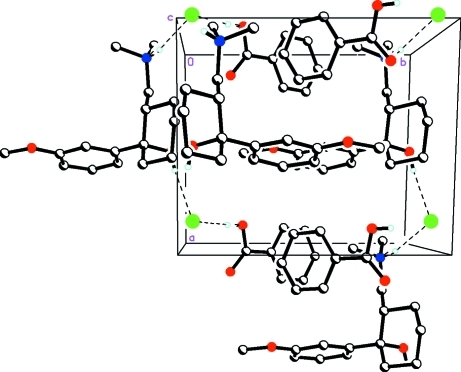
Packing diagram of the title co-crystal salt viewed down the c axis. Dashed lines indicate weak O—H···Cl and N—H···Cl intermolecular interactions forming a supramolecular 2-D chain network along the a axis.

## supplementary crystallographic information

### Comment

Tramadol, chemically, 2-((dimethylamino)methyl)-1-(3-methoxyphenyl) cyclohexanol hydrochloride is classified as a central nervous system drug usually marketed as the hydrochloride salt. Tramadol hydrochloride is a centrally acting opioid analgesic, used in treating moderate to severe pain. The drug has a wide range of applications, including treatment for restless leg syndrome and fibromyalgia. Tramadol is a synthetic analog of the phenanthrene alkaloid codeine and, as such, is an opioid. A review on the use of tramadol in perioperative pain is published (Scott & Perry, 2000). The crystal structures of venlafaxine (Tessler & Goldberg, 2004), benzoic acid-2-{(*E*)-[(*E*)-2-(2-pyridylmethylidene) hydrazin-1-ylidene]methyl}pyridine (2/1) (Arman *et al.*, 2010) and 2,3-diaminopyridinium benzoate benzoic acid solvate (Hemamalini & Fun, 2010) have been reported. In view of the importance of tramadol, this paper reports the crystal structure of a new co-crystal of tramadol hydrochloride with benzoic acid, (I), C_16_H_31_NO_2_^+^ Cl^-^ C_7_O_2_H.

In the cation of title co-crystal salt, (I), the N atom of the amino group is protonated (Fig. 1). The 6-membered cyclohexane group (C4–C9) adopts a slightly distorted chair conformation with puckering parameters Q, θ and φ of 0.5553 (14) Å, 2.55 (14)°, and 271 (3)°, respectively. For an ideal chair θ has a value of 0 or 180°. The dihedral angle between the mean planes of the benzene rings in the cation and the benzoic acid co-crystal is 75.5 (9)°. The crystal packing is stabilized by weak O—H···Cl, N—H···Cl and C—H···*Cg*π-ring intermolecular interactions (Table 1) forming a 2-D chain network along the *a* axis (Fig. 2). The benzoic acid moiety is not involved in the usual head-to-tail dimer bonding, but instead participates in a supramolecular intermolecular interaction between its hydrogen atom and the ammonium cation through the chlorine anion.

### Experimental

Tramadol hydrochloride (2.84 g, 0.01 mol) was dissolved in 10 ml of ethanol and benzoic acid (1.23 g, 0.01 mol) was dissolved in 10 ml of ethanol. Both the solutions were mixed and stirred in a beaker at 333 K for 30 minutes. The mixture was kept aside for three days at room temperature. X-ray quality crystals were formed (m.p: 405–408 K) which was used for data collection.

### Refinement

All of the H atoms were placed in their calculated positions and then refined using the riding model with C—H lengths of 0.95Å (CH), 0.99Å (CH_2_) or 0.98Å (CH_3_) and an N—H distance of 0.93 Å. The isotropic displacement parameters for these atoms were set to 1.2 (CH,  CH_2_, and NH) or 1.50 (CH_3_) times *U*_eq_ of the parent atom.

### Crystal data

**Table:**

C_16_H_26_NO_2_^+^·Cl^−^·C_7_H_6_O_2_	*F*(000) = 452
*M**_r_* = 421.95	*D*_x_ = 1.224 Mg m^−^^3^
Monoclinic, *P**c*	Mo *K*α radiation, λ = 0.71073 Å
Hall symbol: P -2yc	Cell parameters from 8856 reflections
*a* = 8.9721 (2) Å	θ = 3.2–32.3°
*b* = 10.4086 (2) Å	µ = 0.19 mm^−^^1^
*c* = 12.5189 (3) Å	*T* = 173 K
β = 101.646 (2)°	Block, colorless
*V* = 1145.03 (4) Å^3^	0.42 × 0.34 × 0.25 mm
*Z* = 2	

### Data collection

**Table:**

Oxford Diffraction Xcalibur Eos Gemini diffractometer	5354 independent reflections
Radiation source: Enhance (Mo) X-ray Source	5067 reflections with *I* > 2σ(*I*)
graphite	*R*_int_ = 0.014
Detector resolution: 16.1500 pixels mm^-1^	θ_max_ = 28.7°, θ_min_ = 3.2°
ω scans	*h* = −12→12
Absorption correction: multi-scan (*CrysAlis RED*; Oxford Diffraction, 2010)	*k* = −13→14
*T*_min_ = 0.923, *T*_max_ = 0.953	*l* = −16→16
11101 measured reflections	

### Refinement

**Table:**

Refinement on *F*^2^	Secondary atom site location: difference Fourier map
Least-squares matrix: full	Hydrogen site location: inferred from neighbouring sites
*R*[*F*^2^ > 2σ(*F*^2^)] = 0.029	H-atom parameters constrained
*wR*(*F*^2^) = 0.077	*w* = 1/[σ^2^(*F*_o_^2^) + (0.0432*P*)^2^ + 0.0915*P*] where *P* = (*F*_o_^2^ + 2*F*_c_^2^)/3
*S* = 1.04	(Δ/σ)_max_ = 0.001
5354 reflections	Δρ_max_ = 0.21 e Å^−^^3^
267 parameters	Δρ_min_ = −0.14 e Å^−^^3^
2 restraints	Absolute structure: Flack (1983), 2397 Friedel pairs
Primary atom site location: structure-invariant direct methods	Flack parameter: −0.02 (4)

### Special details

**Table:**

Geometry. All e.s.d.'s (except the e.s.d. in the dihedral angle between two l.s. planes) are estimated using the full covariance matrix. The cell e.s.d.'s are taken into account individually in the estimation of e.s.d.'s in distances, angles and torsion angles; correlations between e.s.d.'s in cell parameters are only used when they are defined by crystal symmetry. An approximate (isotropic) treatment of cell e.s.d.'s is used for estimating e.s.d.'s involving l.s. planes.
Refinement. Refinement of *F*^2^ against ALL reflections. The weighted *R*-factor *wR* and goodness of fit *S* are based on *F*^2^, conventional *R*-factors *R* are based on *F*, with *F* set to zero for negative *F*^2^. The threshold expression of *F*^2^ > σ(*F*^2^) is used only for calculating *R*-factors(gt) *etc*. and is not relevant to the choice of reflections for refinement. *R*-factors based on *F*^2^ are statistically about twice as large as those based on *F*, and *R*- factors based on ALL data will be even larger.

### Fractional atomic coordinates and isotropic or equivalent isotropic displacement parameters (Å^2^)

**Table:**

	*x*	*y*	*z*	*U*_iso_*/*U*_eq_	
O1	0.52336 (9)	0.94842 (8)	0.34245 (7)	0.02855 (17)	
H1O	0.6170	0.9644	0.3579	0.034*	
O2	0.50785 (17)	0.37596 (10)	0.40095 (13)	0.0647 (3)	
N1	0.05818 (11)	0.84734 (11)	0.28462 (9)	0.0337 (2)	
H1N	0.0310	0.9119	0.3284	0.040*	
C1	0.0099 (2)	0.72349 (19)	0.32402 (18)	0.0637 (5)	
H1A	−0.1006	0.7241	0.3189	0.096*	
H1B	0.0610	0.7106	0.4002	0.096*	
H1C	0.0371	0.6535	0.2792	0.096*	
C2	−0.02187 (17)	0.87309 (19)	0.17005 (12)	0.0519 (4)	
H2A	−0.1321	0.8698	0.1655	0.078*	
H2B	0.0074	0.8080	0.1216	0.078*	
H2C	0.0066	0.9584	0.1478	0.078*	
C3	0.22703 (13)	0.85528 (12)	0.29216 (10)	0.0298 (2)	
H3A	0.2504	0.9364	0.2577	0.036*	
H3B	0.2586	0.7835	0.2498	0.036*	
C4	0.32126 (12)	0.85020 (11)	0.40805 (9)	0.0254 (2)	
H4A	0.2915	0.7718	0.4449	0.030*	
C5	0.29134 (14)	0.96879 (14)	0.47321 (11)	0.0368 (3)	
H5A	0.3075	1.0471	0.4321	0.044*	
H5B	0.1838	0.9681	0.4812	0.044*	
C6	0.39380 (16)	0.97406 (16)	0.58564 (12)	0.0445 (3)	
H6A	0.3656	0.9037	0.6309	0.053*	
H6B	0.3771	1.0565	0.6209	0.053*	
C7	0.56191 (15)	0.96186 (14)	0.58141 (11)	0.0376 (3)	
H7A	0.5947	1.0390	0.5460	0.045*	
H7B	0.6230	0.9570	0.6566	0.045*	
C8	0.59035 (13)	0.84261 (12)	0.51835 (9)	0.0310 (2)	
H8A	0.5686	0.7653	0.5587	0.037*	
H8B	0.6990	0.8398	0.5134	0.037*	
C9	0.49169 (12)	0.83936 (10)	0.40268 (9)	0.02313 (19)	
C10	0.52315 (12)	0.71504 (10)	0.34583 (9)	0.0254 (2)	
C11	0.50053 (15)	0.59804 (12)	0.39240 (11)	0.0346 (3)	
H11A	0.4621	0.5960	0.4577	0.041*	
C12	0.53321 (16)	0.48312 (13)	0.34504 (13)	0.0405 (3)	
C13	0.58753 (17)	0.48517 (15)	0.24902 (14)	0.0455 (3)	
H13A	0.6103	0.4073	0.2161	0.055*	
C14	0.60801 (17)	0.60191 (16)	0.20207 (12)	0.0458 (3)	
H14A	0.6450	0.6037	0.1361	0.055*	
C15	0.57602 (14)	0.71689 (13)	0.24871 (11)	0.0341 (3)	
H15A	0.5901	0.7963	0.2146	0.041*	
C16	0.5307 (3)	0.25388 (16)	0.3558 (2)	0.0713 (6)	
H16A	0.5050	0.1860	0.4032	0.107*	
H16B	0.6375	0.2453	0.3499	0.107*	
H16C	0.4654	0.2463	0.2832	0.107*	
O3	−0.10582 (14)	1.25355 (13)	0.22279 (12)	0.0598 (3)	
H3O	−0.1056	1.1872	0.2611	0.072*	
O4	0.13609 (18)	1.20037 (17)	0.23538 (18)	0.0883 (5)	
C17	0.03083 (18)	1.27036 (16)	0.20051 (14)	0.0496 (4)	
C18	0.04087 (16)	1.38280 (15)	0.12968 (13)	0.0434 (3)	
C19	−0.08709 (18)	1.44113 (17)	0.06800 (15)	0.0508 (4)	
H19A	−0.1856	1.4105	0.0718	0.061*	
C20	−0.0714 (2)	1.54327 (19)	0.00136 (17)	0.0624 (5)	
H20A	−0.1593	1.5816	−0.0419	0.075*	
C21	0.0714 (2)	1.5907 (2)	−0.00322 (17)	0.0631 (5)	
H21A	0.0813	1.6621	−0.0486	0.076*	
C22	0.1979 (2)	1.53427 (19)	0.05796 (19)	0.0639 (5)	
H22A	0.2960	1.5669	0.0554	0.077*	
C23	0.18366 (19)	1.42998 (19)	0.12347 (17)	0.0584 (4)	
H23A	0.2722	1.3902	0.1646	0.070*	
Cl1	−0.13063 (4)	1.04083 (3)	0.38725 (3)	0.04431 (9)	

### Atomic displacement parameters (Å^2^)

**Table:**

	*U*^11^	*U*^22^	*U*^33^	*U*^12^	*U*^13^	*U*^23^
O1	0.0265 (4)	0.0253 (4)	0.0336 (4)	−0.0005 (3)	0.0054 (3)	0.0049 (3)
O2	0.0903 (9)	0.0227 (5)	0.0842 (9)	0.0030 (5)	0.0248 (7)	0.0023 (5)
N1	0.0243 (4)	0.0418 (6)	0.0331 (5)	0.0007 (4)	0.0013 (4)	−0.0013 (4)
C1	0.0418 (8)	0.0638 (11)	0.0802 (12)	−0.0202 (8)	−0.0003 (8)	0.0176 (9)
C2	0.0380 (7)	0.0747 (11)	0.0371 (7)	0.0095 (7)	−0.0066 (6)	−0.0041 (7)
C3	0.0243 (5)	0.0387 (6)	0.0264 (5)	0.0020 (4)	0.0051 (4)	−0.0016 (5)
C4	0.0224 (4)	0.0293 (5)	0.0248 (5)	−0.0002 (4)	0.0055 (4)	−0.0017 (4)
C5	0.0285 (6)	0.0442 (7)	0.0383 (6)	0.0051 (5)	0.0080 (5)	−0.0136 (6)
C6	0.0397 (7)	0.0594 (9)	0.0345 (7)	0.0020 (6)	0.0076 (6)	−0.0166 (6)
C7	0.0341 (6)	0.0456 (7)	0.0301 (6)	0.0004 (5)	−0.0003 (5)	−0.0103 (5)
C8	0.0293 (5)	0.0337 (6)	0.0272 (5)	0.0034 (4)	−0.0007 (4)	0.0009 (5)
C9	0.0228 (4)	0.0220 (5)	0.0245 (5)	0.0008 (4)	0.0046 (4)	0.0013 (4)
C10	0.0216 (4)	0.0246 (5)	0.0291 (5)	0.0029 (4)	0.0026 (4)	−0.0025 (4)
C11	0.0397 (6)	0.0265 (6)	0.0383 (6)	0.0021 (5)	0.0098 (5)	−0.0007 (5)
C12	0.0404 (7)	0.0253 (6)	0.0527 (8)	0.0039 (5)	0.0022 (6)	−0.0037 (6)
C13	0.0429 (7)	0.0362 (7)	0.0549 (8)	0.0076 (6)	0.0042 (6)	−0.0191 (6)
C14	0.0478 (8)	0.0518 (9)	0.0407 (7)	0.0073 (6)	0.0155 (6)	−0.0121 (6)
C15	0.0347 (6)	0.0343 (6)	0.0347 (6)	0.0026 (5)	0.0107 (5)	−0.0016 (5)
C16	0.0744 (12)	0.0241 (6)	0.1099 (17)	0.0061 (7)	0.0055 (11)	−0.0089 (9)
O3	0.0429 (5)	0.0625 (7)	0.0747 (8)	0.0057 (5)	0.0137 (5)	0.0121 (6)
O4	0.0559 (7)	0.0862 (11)	0.1261 (14)	0.0267 (7)	0.0263 (8)	0.0416 (10)
C17	0.0393 (7)	0.0523 (9)	0.0560 (9)	0.0069 (6)	0.0068 (6)	−0.0057 (7)
C18	0.0351 (6)	0.0461 (8)	0.0477 (8)	0.0028 (5)	0.0056 (6)	−0.0100 (6)
C19	0.0363 (7)	0.0567 (9)	0.0563 (9)	0.0032 (6)	0.0021 (6)	−0.0044 (8)
C20	0.0514 (10)	0.0654 (11)	0.0640 (11)	0.0065 (8)	−0.0039 (8)	0.0028 (9)
C21	0.0745 (12)	0.0568 (10)	0.0585 (10)	−0.0029 (9)	0.0146 (9)	−0.0013 (9)
C22	0.0487 (9)	0.0644 (11)	0.0808 (13)	−0.0068 (8)	0.0181 (9)	−0.0034 (10)
C23	0.0352 (7)	0.0655 (11)	0.0715 (11)	0.0032 (7)	0.0040 (7)	−0.0011 (10)
Cl1	0.02963 (13)	0.05385 (19)	0.05016 (18)	0.00276 (14)	0.00977 (11)	−0.00115 (16)

### Geometric parameters (Å, °)

**Table:**

O1—C9	1.4228 (13)	C8—H8B	0.9900
O1—H1O	0.8400	C9—C10	1.5302 (15)
O2—C12	1.3601 (19)	C10—C11	1.3827 (17)
O2—C16	1.422 (2)	C10—C15	1.3916 (16)
N1—C1	1.476 (2)	C11—C12	1.3923 (18)
N1—C2	1.4930 (17)	C11—H11A	0.9500
N1—C3	1.5010 (14)	C12—C13	1.385 (2)
N1—H1N	0.9300	C13—C14	1.378 (2)
C1—H1A	0.9800	C13—H13A	0.9500
C1—H1B	0.9800	C14—C15	1.3868 (19)
C1—H1C	0.9800	C14—H14A	0.9500
C2—H2A	0.9800	C15—H15A	0.9500
C2—H2B	0.9800	C16—H16A	0.9800
C2—H2C	0.9800	C16—H16B	0.9800
C3—C4	1.5255 (15)	C16—H16C	0.9800
C3—H3A	0.9900	O3—C17	1.323 (2)
C3—H3B	0.9900	O3—H3O	0.8400
C4—C5	1.5329 (16)	O4—C17	1.203 (2)
C4—C9	1.5482 (14)	C17—C18	1.482 (2)
C4—H4A	1.0000	C18—C19	1.387 (2)
C5—C6	1.5188 (19)	C18—C23	1.389 (2)
C5—H5A	0.9900	C19—C20	1.376 (3)
C5—H5B	0.9900	C19—H19A	0.9500
C6—C7	1.5252 (19)	C20—C21	1.384 (3)
C6—H6A	0.9900	C20—H20A	0.9500
C6—H6B	0.9900	C21—C22	1.367 (3)
C7—C8	1.5199 (18)	C21—H21A	0.9500
C7—H7A	0.9900	C22—C23	1.382 (3)
C7—H7B	0.9900	C22—H22A	0.9500
C8—C9	1.5376 (15)	C23—H23A	0.9500
C8—H8A	0.9900		
			
C9—O1—H1O	109.5	C9—C8—H8B	109.1
C12—O2—C16	118.40 (16)	H8A—C8—H8B	107.8
C1—N1—C2	111.16 (13)	O1—C9—C10	110.66 (9)
C1—N1—C3	112.82 (11)	O1—C9—C8	110.06 (9)
C2—N1—C3	109.53 (11)	C10—C9—C8	109.35 (9)
C1—N1—H1N	107.7	O1—C9—C4	105.64 (8)
C2—N1—H1N	107.7	C10—C9—C4	111.00 (8)
C3—N1—H1N	107.7	C8—C9—C4	110.09 (8)
N1—C1—H1A	109.5	C11—C10—C15	119.04 (11)
N1—C1—H1B	109.5	C11—C10—C9	119.48 (10)
H1A—C1—H1B	109.5	C15—C10—C9	121.47 (10)
N1—C1—H1C	109.5	C10—C11—C12	121.05 (12)
H1A—C1—H1C	109.5	C10—C11—H11A	119.5
H1B—C1—H1C	109.5	C12—C11—H11A	119.5
N1—C2—H2A	109.5	O2—C12—C13	125.71 (13)
N1—C2—H2B	109.5	O2—C12—C11	114.46 (13)
H2A—C2—H2B	109.5	C13—C12—C11	119.83 (14)
N1—C2—H2C	109.5	C14—C13—C12	118.96 (13)
H2A—C2—H2C	109.5	C14—C13—H13A	120.5
H2B—C2—H2C	109.5	C12—C13—H13A	120.5
N1—C3—C4	114.61 (9)	C13—C14—C15	121.64 (13)
N1—C3—H3A	108.6	C13—C14—H14A	119.2
C4—C3—H3A	108.6	C15—C14—H14A	119.2
N1—C3—H3B	108.6	C14—C15—C10	119.47 (13)
C4—C3—H3B	108.6	C14—C15—H15A	120.3
H3A—C3—H3B	107.6	C10—C15—H15A	120.3
C3—C4—C5	110.81 (10)	O2—C16—H16A	109.5
C3—C4—C9	108.90 (8)	O2—C16—H16B	109.5
C5—C4—C9	111.25 (9)	H16A—C16—H16B	109.5
C3—C4—H4A	108.6	O2—C16—H16C	109.5
C5—C4—H4A	108.6	H16A—C16—H16C	109.5
C9—C4—H4A	108.6	H16B—C16—H16C	109.5
C6—C5—C4	112.58 (11)	C17—O3—H3O	109.5
C6—C5—H5A	109.1	O4—C17—O3	122.46 (18)
C4—C5—H5A	109.1	O4—C17—C18	123.87 (16)
C6—C5—H5B	109.1	O3—C17—C18	113.67 (13)
C4—C5—H5B	109.1	C19—C18—C23	118.90 (16)
H5A—C5—H5B	107.8	C19—C18—C17	122.35 (14)
C5—C6—C7	112.49 (11)	C23—C18—C17	118.74 (14)
C5—C6—H6A	109.1	C20—C19—C18	120.06 (16)
C7—C6—H6A	109.1	C20—C19—H19A	120.0
C5—C6—H6B	109.1	C18—C19—H19A	120.0
C7—C6—H6B	109.1	C19—C20—C21	120.63 (17)
H6A—C6—H6B	107.8	C19—C20—H20A	119.7
C8—C7—C6	110.91 (11)	C21—C20—H20A	119.7
C8—C7—H7A	109.5	C22—C21—C20	119.60 (19)
C6—C7—H7A	109.5	C22—C21—H21A	120.2
C8—C7—H7B	109.5	C20—C21—H21A	120.2
C6—C7—H7B	109.5	C21—C22—C23	120.27 (18)
H7A—C7—H7B	108.0	C21—C22—H22A	119.9
C7—C8—C9	112.46 (10)	C23—C22—H22A	119.9
C7—C8—H8A	109.1	C22—C23—C18	120.52 (17)
C9—C8—H8A	109.1	C22—C23—H23A	119.7
C7—C8—H8B	109.1	C18—C23—H23A	119.7
			
C1—N1—C3—C4	−64.12 (16)	C15—C10—C11—C12	1.54 (17)
C2—N1—C3—C4	171.50 (12)	C9—C10—C11—C12	−177.65 (11)
N1—C3—C4—C5	−65.45 (13)	C16—O2—C12—C13	−3.5 (2)
N1—C3—C4—C9	171.88 (9)	C16—O2—C12—C11	176.86 (15)
C3—C4—C5—C6	−174.57 (11)	C10—C11—C12—O2	178.89 (13)
C9—C4—C5—C6	−53.27 (14)	C10—C11—C12—C13	−0.8 (2)
C4—C5—C6—C7	53.02 (17)	O2—C12—C13—C14	−179.73 (15)
C5—C6—C7—C8	−53.47 (17)	C11—C12—C13—C14	−0.1 (2)
C6—C7—C8—C9	55.64 (15)	C12—C13—C14—C15	0.2 (2)
C7—C8—C9—O1	59.94 (12)	C13—C14—C15—C10	0.6 (2)
C7—C8—C9—C10	−178.30 (10)	C11—C10—C15—C14	−1.41 (17)
C7—C8—C9—C4	−56.10 (13)	C9—C10—C15—C14	177.77 (11)
C3—C4—C9—O1	57.67 (11)	O4—C17—C18—C19	162.7 (2)
C5—C4—C9—O1	−64.74 (11)	O3—C17—C18—C19	−17.5 (2)
C3—C4—C9—C10	−62.33 (11)	O4—C17—C18—C23	−16.2 (3)
C5—C4—C9—C10	175.26 (10)	O3—C17—C18—C23	163.59 (16)
C3—C4—C9—C8	176.45 (9)	C23—C18—C19—C20	0.5 (3)
C5—C4—C9—C8	54.04 (12)	C17—C18—C19—C20	−178.32 (16)
O1—C9—C10—C11	179.40 (10)	C18—C19—C20—C21	−1.4 (3)
C8—C9—C10—C11	58.00 (13)	C19—C20—C21—C22	0.9 (3)
C4—C9—C10—C11	−63.64 (13)	C20—C21—C22—C23	0.4 (3)
O1—C9—C10—C15	0.22 (14)	C21—C22—C23—C18	−1.3 (3)
C8—C9—C10—C15	−121.17 (11)	C19—C18—C23—C22	0.8 (3)
C4—C9—C10—C15	117.18 (11)	C17—C18—C23—C22	179.69 (16)

### Hydrogen-bond geometry (Å, °)

**Table:**

Cg3 is the centroid of the C18–C23 ring.

**Table:**

*D*—H···*A*	*D*—H	H···*A*	*D*···*A*	*D*—H···*A*
O1—H1O···Cl1^i^	0.84	2.36	3.1898 (9)	171.
O3—H3O···Cl1	0.84	2.24	3.0620 (14)	167.
N1—H1N···Cl1	0.93	2.21	3.0750 (12)	155.
C1—H1B···Cg3^ii^	0.98	2.90	3.662 (5)	135

Symmetry codes: (i) *x*+1, *y*, *z*; (ii) *x*, −*y*+1, *z*+1/2.
